# Synchrotron‐Based Analysis of Conical Implant–Abutment Connections Under Mechanical Load: How Embedding Materials Influence the Microgap and Implant Shoulder Deformation

**DOI:** 10.1002/cre2.70179

**Published:** 2025-08-14

**Authors:** Florian Kernen, Katja Nelson, Simon Zabler, Alexander Rack, Fumihiko Watanabe, Tobias Fretwurst, Sina Wenger

**Affiliations:** ^1^ Department of Oral‐ and Maxillofacial Surgery University Medical Center, Faculty of Medicine, University of Freiburg Germany; ^2^ Deggendorf Institute of Technology DIT Deggendorf Germany; ^3^ European Synchrotron Radiation Facility – ESRF Grenoble Cedex 9 France; ^4^ Department of Crown & Bridge Prosthodontics, School of Life at Niigata The Nippon Dental University Japan; ^5^ Department of Oral Medicine Infection, and Immunity, Harvard School of Dental Medicine Boston Massachusetts USA

**Keywords:** dental implant, embedding material, mechanical behavior, micro gap

## Abstract

**Objectives:**

Dental implants have become a reliable solution for oral rehabilitation, but their long‐term success can be compromised by factors such as mechanical overload. To ensure the mechanical durability of implants, standardized testing in accordance with the DIN EN ISO 14801 is conducted, in which titanium implants (Young's modulus of approximately 100 GPa) are embedded in brass, a material with similarly high stiffness. However, the mechanical properties of brass differ significantly from those of alveolar bone, potentially affecting test outcomes. This study examines how different embedding materials affect the mechanical behavior of dental implants, specifically microgap changes and deformation at the IAC under load.

**Materials and Methods:**

Two conical dental implants were embedded in either methyl methacrylate‐based adhesive (PMMA) or brass. A 250 N load was applied at a 45° angle to the implants. Synchrotron‐based microcomputed tomography (µCT) was used to assess microgap formation and 3D deformation at the implant shoulder before and under load application. Deformation was analyzed using Avizo Fire software to estimate volumetric changes at the implant shoulder.

**Results:**

The results showed that implants embedded in brass exhibited larger microgap changes (53 μm) and greater deformation at the implant shoulder (32 μm) compared to those embedded in PMMA (microgap: 40 μm).

**Conclusion:**

The findings suggest that brass, with higher stiffness than PMMA or bone, does not accurately replicate the mechanical conditions of bone, leading to a difference in microgap behavior and deformation at the implant shoulder, suggesting a difference in the wear mechanism and stress‐strain distribution in the surrounding bone. These results question the use of brass in mechanical implant testing and highlight the need for more realistic embedding materials to improve the predictive value of implant testing.

## 1 Introduction

In recent decades, dental implants have emerged as a reliable option for oral rehabilitation, demonstrating high survival rates and favorable long‐term outcomes (Buser and Tonetti 1997). However, several risk factors may compromise the long‐term success of implants. Key factors include inadequate oral hygiene and periodontitis, along with the biomechanical behavior of the implant, which can contribute to peri‐implant mucositis and peri‐implantitis (Smeets et al. 2014; Belibasakis and Manoil 2021; Fretwurst et al. 2016). Additionally, preclinical studies have indicated that occlusal trauma and mechanical overload may lead to progressive bone loss or even complete loss of osseointegration around implants that were previously considered fully osseointegrated (Chang et al. 2013; Mattheos et al. 2012; Isidor 1997).

Furthermore, it has been suggested that cyclic loading at the implant–abutment interface may cause wear, leading to the release of particles and ions into the surrounding peri‐implant soft and hard tissues and potentially contributing to bone loss (Fretwurst et al. 2016, 2018; Nelson 2020). Based on a series of preclinical studies employing synchrotron analysis, the microenvironment and deformation of the implant–abutment connection (IAC) under load have been characterized. Using synchrotron‐based X‐rays enables the investigation of structures and properties of materials at the submicrometer level, revealing elastic and plastic deformation of the implant shoulder, which may generate stress and damage to the surrounding bone, depending on the amplitude and frequency of the applied forces (Nelson et al. 2016; Angermair 2024; Duyck and Vandamme 2014; Ogawa 2011; Naert et al. 2012; Blum 2015).

Mechanical testing of dental implants in vitro, particularly for novel designs, is essential before clinical implementation. To ensure the mechanical durability of an implant and to evaluate its resistance to functional load applications, standardized testing in accordance with the DIN EN ISO 14801 is conducted (Marchetti 2016). During this procedure, implants are mounted within a brass cylinder and exposed to cyclical force to the implant (Brandes et al. 1998). The implants are positioned to protrude 3 mm from the embedding material, simulating bone resorption around the implant. Failure of the system is defined as any of the following: material yielding, permanent deformation, loosening of the implant assembly, and fracture of any implant component (DIN EN ISO 14801:2017‐03) (Dittmer et al. 2011).

The fracture toughness of metals is assessed following standards such as American ASTM E399‐83 or British BS 5447:1977. In these standard testing methodologies, metal specimens are subjected to incrementally increasing static forces until the material transitions from elastic to plastic deformation and ultimately fails (Brandes et al. 1998). Failure, as defined by ISO 14801:2017‐03, occurs when a sudden drop in force or a halt in displacement is recorded during increased load application, as evidenced by a load‐displacement curve. This analysis captures crack initiation or growth as well as plastic deformation before fracture. Notably, optical or radiographic inspections of the tested assembly are not required. Additionally, the tests do not account for the properties of the embedding material or its influence on the behavior of the IAC. The use of excessively rigid embedding materials may lead to artificially elevated loads on the IAC, potentially resulting in an overestimation of microgap under these conditions since the Young's modulus of bone ranges from 5 to 22 GPa, depending on its microstructure (cancellous vs. cortical bone) (Rho et al. 1998; Wu et al. 2018).

Collectively, these factors suggest that current testing methodologies may not adequately address the clinical implications, particularly given that excessive loading on bone can lead to resorption (Bertolini et al. 2019; de Calais et al. 2021). Moreover, visual assessments during testing do not provide insights into the spatial interactions of the implant, abutment, microgap, or the extent of deformation and wear. While laboratory‐based X‐ray imaging offers geometric information non‐destructively, synchrotron‐based imaging, particularly microcomputed tomography (µCT), provides superior material contrast and higher spatial resolution compared to conventional laboratory CT (Blum 2015; Zabler et al. 2010). The µCT imaging is complemented by three‐dimensional (3D) deformation analysis, allowing for the evaluation of diffraction patterns of synchrotron radiation as it interacts with the material to assess its 3D deformation (Bagegni 2021). The null hypothesis (N_0_) of this study is that the embedding material influences and even distorts the results of the mechanical load tests in in vitro studies.

## 2 Methods

### 2.1 Sample Preparation and Force Application

**Figure 1:**
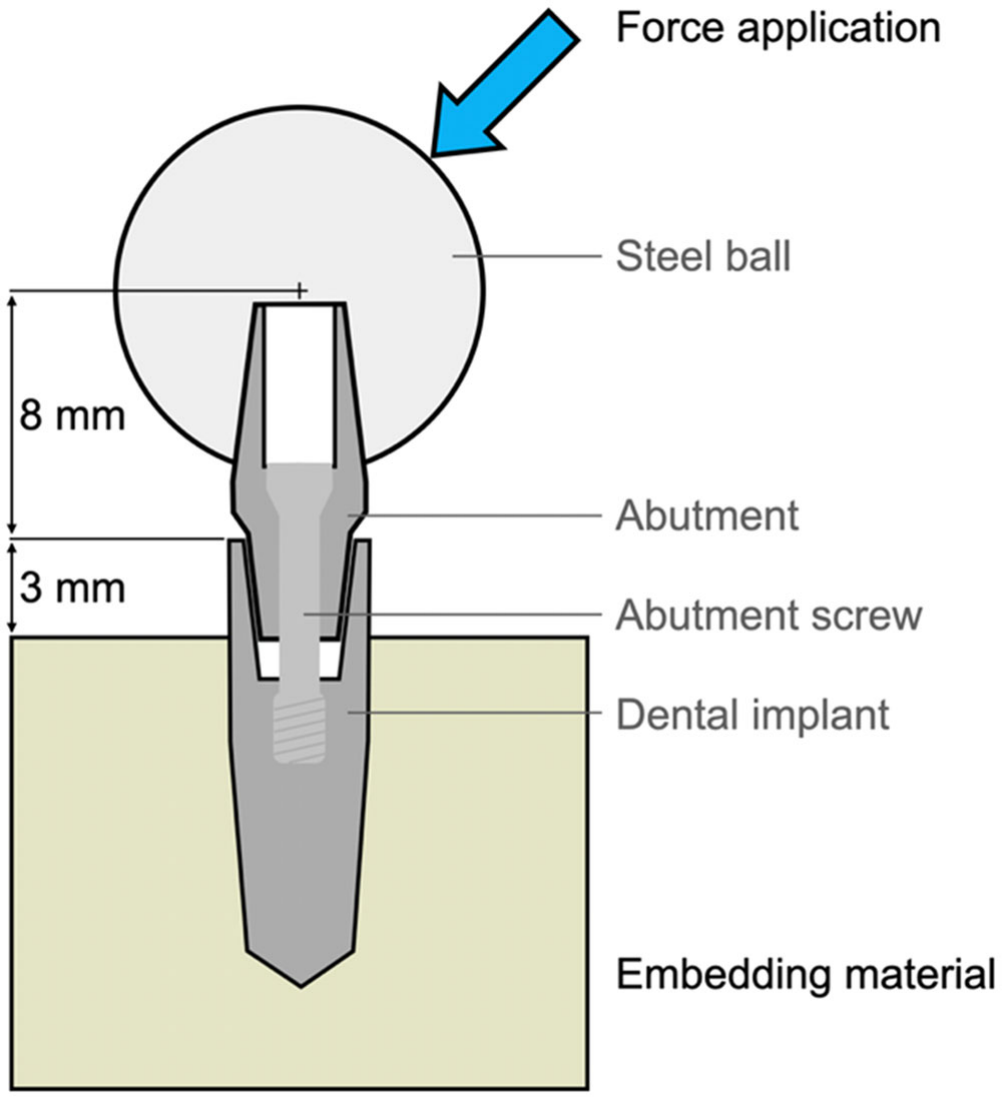
Schematic setup based on the DIN EN ISO standard. In contrast to the standard, different embedding materials (PMMA and brass) were used in our experiment.

The mechanical behavior in relation to the embedding material of two conical implants of identical system design (CONELOG SCREW‐LINE Promote Implants, 3.8 × 11 mm, Camlog, Switzerland) was tested with synchrotron‐based analysis in this technical note (Table 1). Therefore, one implant was fully embedded in a methyl methacrylate‐based adhesive (PMMA, X60, Germany), while the control implant was rigidly glued (X60, Germany) in a pre‐drilled brass cylinder according to DIN EN ISO 14801:2017‐03 (Dittmer et al. 2011). Two abutments were screw tightened with a system‐specific torque (Conelog Abutment screw, Camlog, Switzerland; 20 Ncm) according to the manufacturer's guidelines, and a 10 × 10 mm steel ball was attached (X60, HBM Germany) to the abutments to allow off‐axis force application. A single load application of 250 N at a 45° angle relative to the long axis of the implant was applied using a controlled force gauge (SH‐500, PCE‐group OHG, Germany) on the experimental setup, and measurements were performed before and under load application (Figure 1).

**Table 1:** Geometric data of the tested implant system and abutments.

Detailed implant data	
Implant system	CONELOG SCREW‐LINE Promote, Camlog, Switzerland
Type of connection	Conical
Cone angle [°]	7.5
Implant diameter [mm]	3.8
Implant length [mm]	11
Detailed abutment data	
Abutment system	CONELOG Abutment screw, Camlog, Switzerland
Abutment diameter [°]	9

### 2.2 Synchrotron‐Based Microtomography

The deviation in the microgap before and under load application on the embedded implants, influenced by the embedding material, was analyzed using synchrotron‐based methods. Synchrotron µCT scans of dental implants were performed at the BAMline on the BESSY‐II light source in Berlin, Germany (X‐ray wavelength: 24.8 pm, vertical beam‐size: 1.4 mm, horizontal beam‐size: 20 mm). To achieve propagation‐based phase contrast, the indirect detector (“Optique Peter”, Lentilly, France) was positioned 1000 mm downstream of the samples. The detector employed a CCD‐Camera (PCO 4000, PCO GmbH, Germany) in conjunction with a 50‐µm‐thick Cadmium Tungstate scintillator, with a detector pixel sampling of 2.17 µm.

### 2.3 Assessment of the Microgap

Quantitative values of the microgap size were estimated by comparing the line profile of the phase contrasted IAC as described previously (Nelson et al. 2016; Zabler et al. 2010). Additionally, the radial width of the conical microgap over a 360° implant radius was inspected by virtual cylindrical projections. The latter allows for comparing the abutment's displacement at every point on the IAC (Blum 2015; Zabler et al. 2010).

### 2.4 Deformation of the Implant Shoulder

To analyze 3D deformation, synchrotron µCT scans before and under load application were superimposed. Because threshold application does not yield an automated segmentation of implant, abutment, and abutment screw, semi‐manual segmentation was applied using the watershed algorithm and identifying connected components (Rho et al. 1998). From these three parts, volumetric surfaces were generated, which were used to estimate volumetric deformation using the software Avizo Fire (Thermo Fisher GmbH).

## 3 Results

### 3.1 Microgap

**Figure 2:**
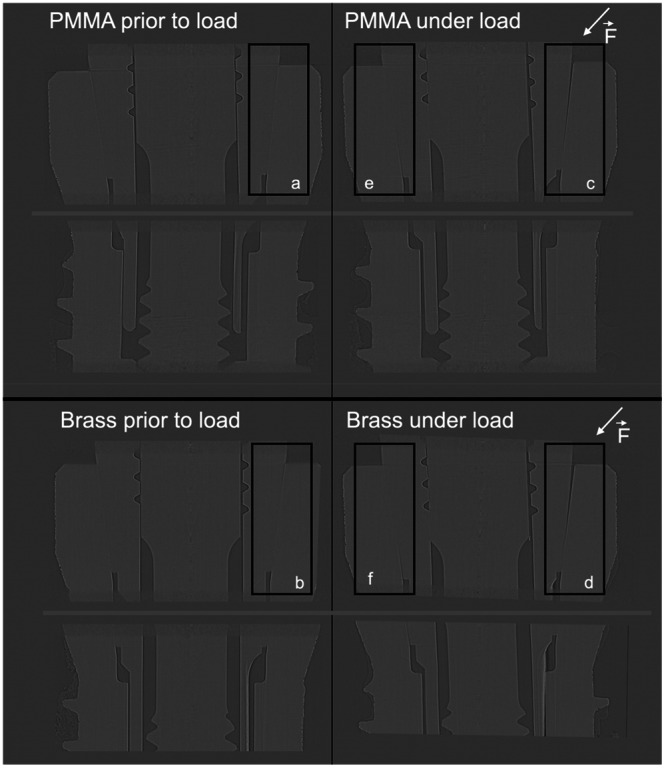
Assessment of microgap formation of dental implants embedded in PMMA versus brass using synchrotron‐based microcomputed tomography (µCT): (a). 8 µm, (b). 7 µm (before load), (c). 40 µm, (d). 53 µm, (e). 14 µm, (f). 19 µm (under load application). X‐ray wavelength: 24.8 pm, vertical beam size: 1.4 mm, and horizontal beam size: 20 mm before and under load application with 250 µm and 45° force angle.

**Figure 3:**
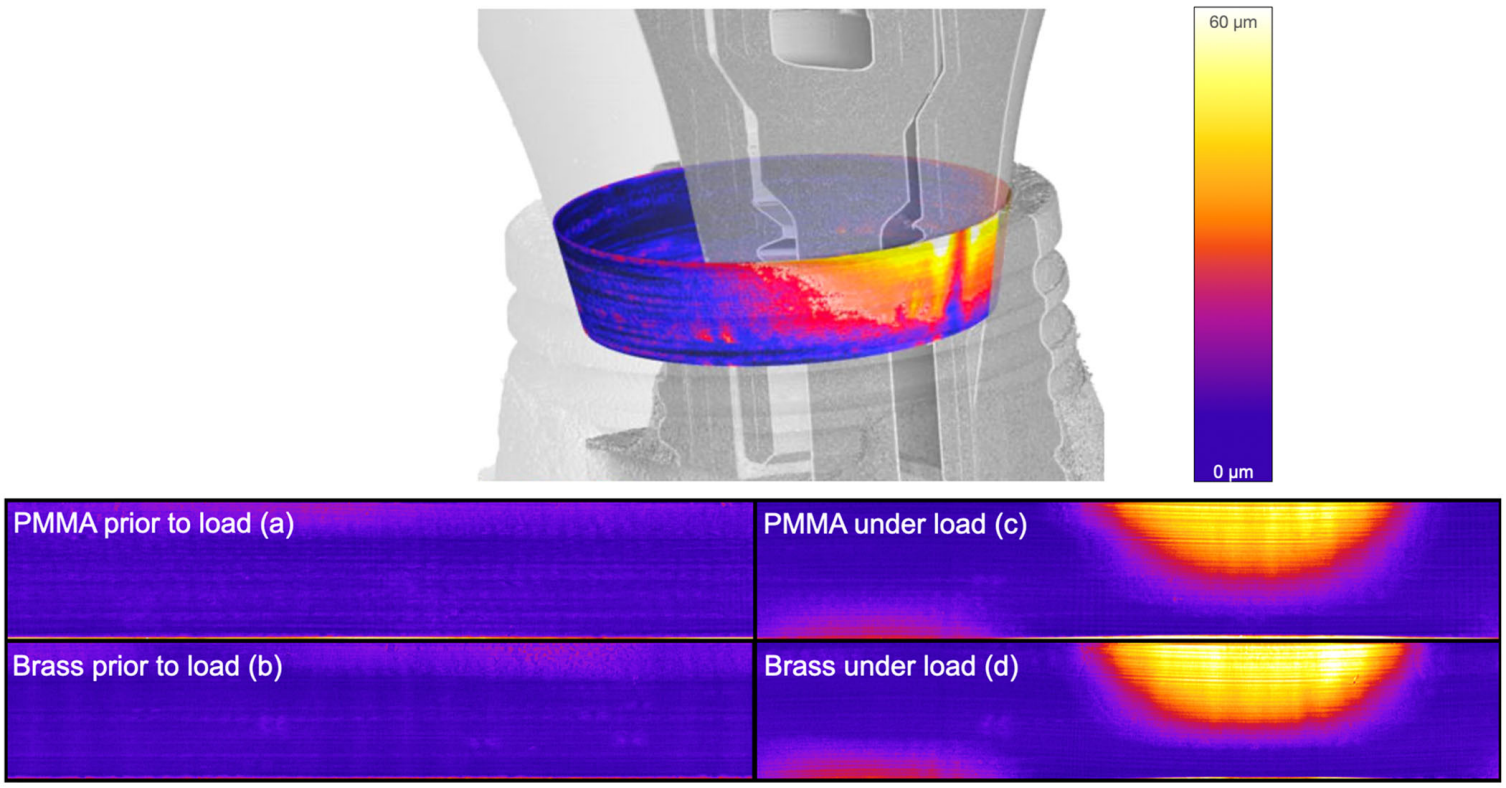
Visualization of abutment inclination and microgap formation of dental implants embedded in PMMA compared to brass before and under loading at 250 µm and 45° with a gap map from purple to white with increasing gap (0 µm to approximately 60 µm). The second micro‐gap at the bottom left is the result of the abutment tilting, which leads to the formation of a second gap on the opposite side.

Before load, both implants showed a microgap at the IAC of 8 μm in PMMA (a) and 7 μm in brass (b) as previously described (Angermair 2024). When a force of 250 N was applied to the steel ball at a 45° angle, a V‐shaped enlargement of the microgap was observed in both implant systems, which can be attributed to a tilting of the abutment under the force. A smaller increase of the microgap at the IAC was measured in the implant embedded in PMMA, with 40 μm (c), compared to 53 μm (d) for the implant embedded in brass on the force‐applying side. Due to the tilting of the abutment, a secondary micro‐gap formation was observed deeper on the side opposite to the applied force. The IAC embedded in PMMA exhibited a secondary microgap of 14 μm (e), while the gap was larger in brass, measuring 19 μm (Figure 2). The expansion of the microgap was visualized using a color scale, increasing in size from blue to yellow. With this analysis, line profile data were visualized in 3D. The color scale further verified visually that the gap expansion was larger when the IAC was embedded in PMMA compared to brass (Figure 3).

### 3.2 Three‐Dimensional Deformation

**Figure 4:**
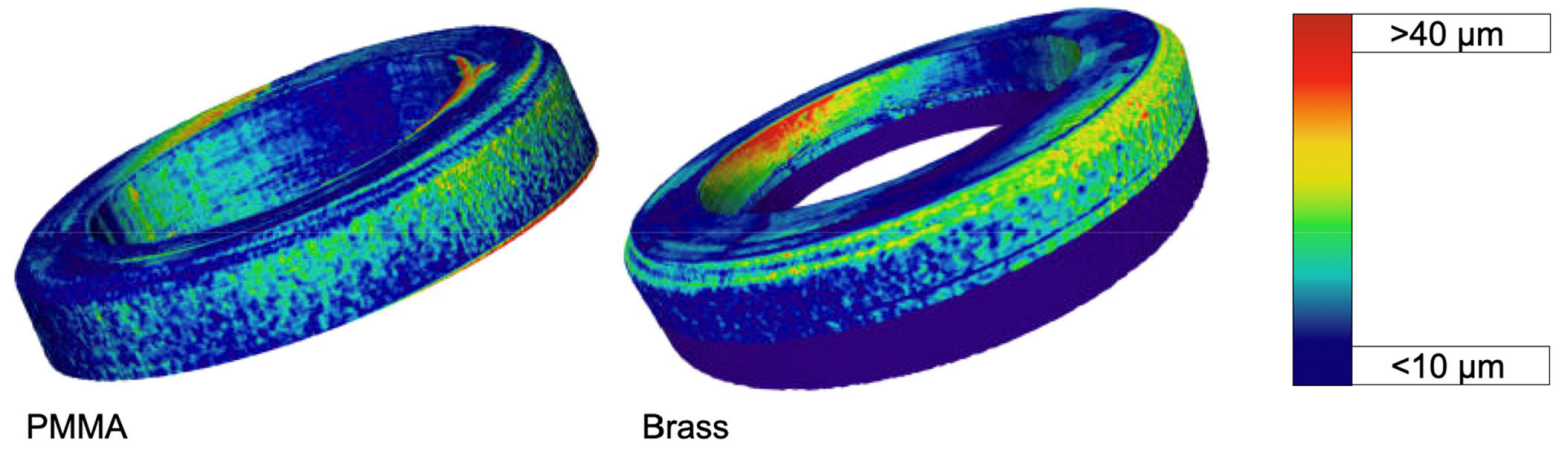
3D deformation analysis of the implant shoulder under load application of 250 N at an angle of 45° visualized with a heat map (ranging from blue (< 10 µm) to red (> 40 µm) with increasing deformation pattern). The deformation of the implant embedded in PMMA (left) displayed less deformation of the inner implant shoulder than the implant embedded in the brass (right). Values for the deformation of the inner implant shoulder reached 20 µm embedded in PMMA and up to 32 µm for the brass‐embedding.

An additional analysis was conducted to examine the deformation at the inner implant shoulder in relation to the embedding material under force application to the IAC. Under force application, both implants ‐ whether embedded in PMMA or brass ‐ exhibited deformation at the implant shoulder on the contralateral side of the applied force. The implant embedded in brass displayed a greater deflection, as visualized on the color scale. In this case, the deformation extended into the maximum range, which is approximately 32 μm (Figure 4) compared to the PMMA‐embedding with approximately 18 μm.

## 4 Conclusion

The present in‐vitro study demonstrated that the choice of embedding material in mechanical testing of dental implants significantly impacts the observed mechanical behavior.

The observed microgaps at IACs embedded in brass and PMMA before load application ranged from 7 to 9 μm, consistent with previous studies (Angermair 2024). After load application, the widening of the microgap at the IAC was more pronounced in brass‐embedded compared to PMMA‐embedded implants (53 vs. 40 μm), while previous studies on brass‐embedded conical implants reported similar microgap behaviors, the response of PMMA‐embedded implants had not been studied before (Nelson et al. 2016; Naert et al. 2012; Zabler et al. 2010).

Many studies investigated the influence of loading forces on microgap formation at the implant–abutment connection (IAC). Angermair et al. measured IACs under varying loads, which clearly influenced microgap behavior. Under a horizontal force of 30 N, microgaps developed predominantly at the side of load application, with the widest openings observed in a conical IAC (0.6–15 μm), followed by a butt‐joint connection (7–11 μm). When the load increased to 100 N, all implant systems exhibited significantly larger microgaps, with maximum values reaching 40.5 μm in butt‐joint IACs. In conical connections, the load‐induced displacement resulted not only in asymmetric microgap widening but also in abutment tilting within the IAC. Notably, at 200 N applied at 30°, conical implants showed plastic deformation of the implant shoulder and persistent microgap formation, emphasizing the mechanical limitations of certain systems under excessive functional loading (Angermair 2024).

The expansion of the IAC microgap indicated a tilting of the abutment under load, a phenomenon previously reported. An increasing force was applied to various brass‐embedded conical and butt‐joint IACs, resulting in tilting of the abutment relative to the implant in all systems tested. For conical IACs, the angle of the mating zone influenced the geometry of the microgap. For example, small angles, e.g., 7.5°, as the angle of the implants used in this study, resulted in a V‐shaped gap, while conical mating zones with larger angles (> 10°) resulted in a nearly parallel gap opening (Angermair 2024; Rack et al. 2010). However, it is important to note that the extent of microgap expansion varied in this study, even though similar implants were tested, indicating the influence of the embedding material on microgap behavior. These behavioral differences highlight that the type of micromotion of the abutment is influenced by its environment.

Brass, with its high stiffness and comparable Young's modulus to titanium (approximately 100 GPa), resists deformation more than PMMA. As a result, more force is transferred directly to the IAC, causing greater deformation at the connection. In contrast, PMMA exhibits greater elasticity, allowing a displacement of the implant and the abutment in the embedding‐material under load. This facilitates energy dissipation into the embedding material, which reduces the force transmitted to the IAC. Consequently, the displacement at the IAC is smaller, suggesting PMMA better mimics the mechanical behavior of bone, potentially offering a more accurate reflection of in vivo conditions.

The deformation of the implant shoulder under load application has been described previously using synchrotron imaging (Nelson et al. 2016; Zabler et al. 2012). Within this study, the plastic deformation of the implant shoulder on the opposite side of load application amounted up to 32 µm when the implant was embedded in brass. In contrast, the deformation of the implant shoulder was lower, indicating a deflection of the embedding material due to its elasticity. These findings verify previous data from Zabler et al. which investigated the mechanical deformation behavior of the IAC using X‐ray phase contrast microtomography. This study detected plastic deformation of the IAC under cyclic load, with variations across different implant systems (Zabler et al. 2012). These findings suggest that not only the gap behavior is affected by the embedding material, but also the distribution and magnitude of forces transmitted to both the entire IAC and the embedding material. Given that PMMA has a similar Young's modulus as bone, it is reasonable to assume that this material more closely replicates physiological conditions, which results in less deformation at the implant shoulder. Due to these findings, the question arises as to whether the brass embedding, with its more extreme deformation patterns, might actually provide a more reliable model for mechanical testing, potentially ensuring greater safety in clinical applications.

It is important to note that the mechanical behavior of the IAC changes depending on the embedding material. When embedded in PMMA, a tilting of the abutment against the implant shoulder was measured, leading to deflection of the IAC and embedding‐material‐complex as the force is transferred into the embedding material. In contrast, when embedded in brass, no deflection was seen. This phenomenon results in greater particle wear at the IAC when embedded in brass compared to PMMA. Blum et al. demonstrated, using synchrotron imaging, that titanium particle abrasion can occur under (cyclic) load at the IAC. The main wear mechanisms that occur along the mating surface of metal‐to‐metal joints are tribochemical reactions that deposit a mixture of wear particles, metal ions, and organic matrix of decomposed proteins named tribolayer. These particles accumulate in the tribolayer in various shapes and sizes. In the study by Blum et al. the implants were first embedded in PMMA for 200,000 chewing cycles, followed by 800,000 cycles re‐embedded in brass. A difference in particle formation and accumulation was observed in implants embedded in brass compared to PMMA, suggesting a difference in force distribution in the IAC and therefore wear behavior (Blum 2015). While the implant embedded in brass showed increased particle abrasion, PMMA embedded implants exhibited less abrasion.

Titanium is regarded as an ideal biomaterial for implants due to its excellent biocompatibility, osseointegration, corrosion resistance, and mechanical properties (Nicholson 2020). It is widely used in dental and maxillofacial surgery. However, titanium can be damaged by machining, friction, mechanical wear, and corrosion enhanced by cellular processes over time, leading to the release of Ti particles. Particles have been found in tissue of failing implants; their role in the emergence and/or progression of peri‐implant disease is unknown (Fretwurst et al. 2018). The immunological pathways induced depend on the cellular uptake and translocation of micro‐ and nanoparticles, which depends on the size, shape, and surface chemistry of the particles (Nelson 2020; Bertolini et al. 2019; Sarraf et al. 2022). Knowledge of the wear and deformation mechanism of IACs can be essential to understand the development and progression of peri‐implant disease.

The differences in the degree of the deformation of the outer implant shoulder in regard to the embedding medium suggest a variation in the stress‐strain distribution within the system abutment, implant, and bone. Bone adaptation to mechanical stress and strain is essential for maintaining its integrity under physiological loading conditions (Becker 2021). However, strain‐induced damage can lead to bone resorption. Despite this understanding, there is a lack of experimental data mimicking clinical settings that quantitatively assess how stress and strain are transmitted into the surrounding bone. Animal studies suggest that efficient force transfer may promote bone formation and osseointegration (Duyck and Vandamme 2014). Several factors influence the stress‐strain distribution, including implant and abutment diameter, material properties, IAC design, as well as bone architecture, physiology, and loading angles. Further research is needed to explore these parameters in greater depth to optimize implant designs and predict bone responses more accurately in clinical practice.

The results of this study enable new perspectives on the standardization of mechanical testing in dental implants. While brass provides consistency and durability in experimental setups, its mechanical properties do not replicate those of human bone. This discrepancy highlights the need to reconsider the choice of embedding materials for a more realistic depiction of clinical scenarios.

Furthermore, the findings suggest a causal relationship between the stiffness of the embedding material and the stress‐strain distribution within the implant.

While this study provides strong evidence of this relationship, further research is required to validate these findings. In particular, testing larger sample sizes and incorporating osseointegrated IACs from human donors could lead to a deeper understanding of the interaction between embedding material properties and implant biomechanics, ultimately improving the reliability and relevance of mechanical testing in dental implantology. Additionally, these findings could improve Finite Element Analysis (FE), which is a computational technique used to simulate and analyze the mechanical behavior of dental implants and surrounding tissues under different loading conditions. In the context of dental implants, FE allows to evaluate the stress distribution, strain, and deformation of implants, abutments, and bone, which are critical factors in the design and optimization of implant systems (Wiest et al. 2018).

In conclusion, the mechanical behavior of implants under load depends on the embedding medium. Implants embedded in brass demonstrated different microgap behavior and a greater deformation of the implant shoulder compared to those embedded in PMMA, suggesting a difference in the stress‐strain distribution. To understand the stress–strain distribution in the bone surrounding the implant materials that closely mimic the properties of bone is essential to replicate physiological conditions.

## Author Contributions

Sina Wenger, Florian Kernen, Simon Zabler, Alexander Rack, and Katja Nelson acquired the data. Sina Wenger, Florian Kernen, Tobias Fretwurst, and Katja Nelson wrote the manuscript. Sina Wenger, Florian Kernen, Simon Zabler, Alexander Rack, Fumihiko Watanabe, Tobias Fretwurst, and Katja Nelson revised the manuscript.

## Ethics Statement

The authors have nothing to report.

## Consent

The authors have nothing to report.

## Conflicts of Interest

The authors declare no conflicts of interest.

## Data Availability

The data that support the findings of this study are available from the corresponding author upon reasonable request.
